# Clinical Benefit from Lenvatinib and Pembrolizumab Observed in Mullerian Adenosarcoma: A Case Report

**DOI:** 10.3390/curroncol28030199

**Published:** 2021-06-10

**Authors:** Thierry Alcindor, Sungmi Jung, Lucy Gilbert

**Affiliations:** 1 Cedars Cancer Center, Division of Medical Oncology, McGill University Health Centre, 1001 Decarie Boulevard, Montreal, QC H4A 3J1, Canada; 2 Department of Pathology, McGill University Health Centre, 1001 Decarie Boulevard, Montreal, QC H4A 3J1, Canada; sungmi.jung@mcgill.ca; 3 Cedars Cancer Center, Division of Gynecologic Oncology, McGill University Health Centre, 1001 Decarie Boulevard, Montreal, QC H4A 3J1, Canada; lucy.gilbert@mcgill.ca

**Keywords:** adenosarcoma, sarcoma, immunotherapy, targeted therapy

## Abstract

A 32-year-old woman with chemorefractory mullerian adenosarcoma showed clinical benefit in response to administration of lenvatinib plus pembrolizumab. In this case report, we describe the course of her illness and her response to this treatment.

## 1. Introduction

Mullerian adenosarcoma is a rare gynecological cancer characterized by a biphasal aspect, consisting of a benign epithelial proliferation in addition to a malignant stroma [1]. While low-grade tumors have an indolent course, the presence of sarcomatous overgrowth, defined as 25% or more of stromal component, confers clinical aggressiveness [2]. These cancers tend to have little sensitivity to systemic therapy [3]. Little has been published about their biology, which may explain the rarity of hypothesis-driven trials dedicated to them. To our knowledge, immunotherapy and molecularly targeted therapy have not been systematically investigated in this group of tumors. We present here what we believe to be the first case of mullerian adenosarcoma with sarcomatous overgrowth that benefited from lenvatinib and pembrolizumab, a treatment recently approved for endometrial adenocarcinoma.

## 2. Case Description

A 32-year-old primiparous woman sought medical attention for the appearance of a rapidly enlarging and painful pelvic mass 6 months after vaginal delivery, accompanied by irregular uterine bleeding. She had a history of infertility attributed to endometriosis and treated with hormonotherapy (dienogest). She was a nonsmoker and had no history of drug or alcohol abuse. The physical examination showed a woman measuring 160 cm with a weight of 85 kg, approximately. Imaging studies revealed a large abdominopelvic mass of malignant appearance with no lymphadenopathy and no distant metastases. During preparation for elective surgery, she presented for severe abdominal pain to the Emergency Department where the diagnosis of an intraabdominal perforation was entertained. An urgent laparotomy was immediately performed, resulting in the palliative resection and debulking of a perforated mass of pelvic origin with extensive involvement of the abdominal cavity.

The pathology report described a 24 cm tumor with honeycomb appearance and cystic degeneration, made up of benign mullerian-type glands admixed with a malignant stromal/mesenchymal hypermitotic and cytologically atypical component, diagnostic of mullerian adenosarcoma with extensive high-grade sarcomatous stromal overgrowth and without heterologous components. The malignant cells stained with ER, PR, cyclin D1 and WT1, suggestive of a gynecologic origin. It was not possible to determine whether the primary site was uterine or extra-uterine. Immunohistochemistry showed no mismatch repair (MMR) protein loss. An inhouse next-generation sequencing platform of the tumor designed to detect DNA and RNA alterations in 52 genes (Table 1) only revealed a *KRAS* mutation (p.Gly12Asp). PD-L1 expression on tumor cells was 0%.

The patient was started on doxorubicin for residual disease and received six cycles, which resulted in disease stabilization, as best response. Radiotherapy also had to be given for a bleeding intra-rectal lesion. At progression, chemotherapy was changed to gemcitabine/docetaxel, with disease stabilization; this treatment had to be stopped for toxicity (fatigue and thrombocytopenia) after 4 months. Stability was maintained for another 5 months with anastrozole. For major disease progression with the appearance of new lesions and partial bowel obstruction, ifosfamide was given but, despite short-term stabilization, did not prevent further progression. On the basis of the success of lenvatinib/pembrolizumab in endometrial cancer, this regimen was prescribed to the patient after informed consent through a compassionate program. A clinically meaningful tumor reduction was observed, although not qualifying for partial response per RECIST criteria, after 9 weeks of treatment. Regrowth of a tumor implant was seen after 18 weeks, which did not respond to tumor embolization. Frank disease progression was evident with new tumor deposits after 8 months of lenvatinib/pembrolizumab. Two cycles of trabectedin were unsuccessful and the patient died after opting for palliative care. Survival was 29 months between surgery and death. The various tumor sizes through treatment stages are listed in Table 2.

## 3. Discussion and Conclusions

Adenosarcoma and endometrial adenocarcinoma are considered separate diseases, although endometrioid carcinoma mixed with clonally related adenosarcoma is reported [4]. Lenvatinib/pembrolizumab is active in metastatic or unresectable endometrial adenocarcinoma, despite low prevalence of PD-L1 expression [5] and regardless of MMR protein status [6]. Although our patient’s tumor did not have any adenocarcinoma component, given the lack of standard therapeutic options and the absence of any suitable clinical trial in our institution, this regimen was offered to her on the basis of the known sensitivity of various sarcomas to antiangiogenic tyrosine kinase inhibitors [7,8] and of the synergy observed between the latter and immune checkpoint inhibitors [9]. This regimen has not been specifically studied in gynecological sarcomas to our knowledge. Its activity in our patient suggests that it would be worthy of a prospective investigation in mullerian adenosarcoma. In fact, PD-L1 expression, a predictive factor of response to immune checkpoint inhibition, although absent in our case, has been reported in a subset of uterine adenosarcomas [10].

## Figures and Tables

**Table 1:** Genes tested in inhouse panel. Only a *KRAS* mutation was found. *TP53* was not tested.

Gene Mutations	Copy-Number Variants	Gene Fusions
*AKT1*	*ALK*	*ABL1*
*ALK*	*AR*	*ALK*
*AR*	*BRAF*	*AKT3*
*BRAF*	*CCND1*	*AXL*
*CDK4*	*CDK4*	*BRAF*
*CTNNB1*	*CDK6*	*EGFR*
*DDR2*	*EGFR*	*ERBB2*
*EGFR*	*ERBB2*	*ERG*
*ERBB2*	*FGFR1*	*ETV1*
*ERBB3*	*FGFR2*	*ETV4*
*ERBB4*	*FGFR3*	*ETV5*
*ESR1*	*FGFR4*	*FGFR1*
*FGFR2*	*KIT*	*FGFR2*
*FGFR3*	*KRAS*	*FGFR3*
*GNA11*	*MET*	*MET*
*GNAQ*	*MYC*	*NTRK1*
*HRAS*	*MYCN*	*NTRK2*
*IDH1*	*PDGFRA*	*NTRK3*
*IDH2*	*PIK3CA*	*PDGFRA*
*JAK1*		*PPARG*
*JAK2*		*RAF1*
*JAK3*		*RET*
*KIT*		*ROS1*
*KRAS*		
*MAP2K1*		
*MAP2K2*		
*MET*		
*MTOR*		
*NRAS*		
*PDGFRA*		
*PIK3CA*		
*RAF1*		
*RET*		
*ROS1*		
*SMO*		

**Table 2:** Sequence of treatments.

Treatment Regimen	Dose and Treatment Frequency	Duration of Treatment	Best Response Observed	Tumor Size at Start	Tumor Size When Treatment Stopped	Comments
Doxorubicin	75 mg IV/m^2^ every 3 weeks	4 months (5 cycles)	SD	A: 4.7 × 3.5 cm	A: 6 × 4 cm B: 2.4 × 2.6 cm	Stopped for progression
Gemcitabine + Docetaxel	900 mg/m^2^ (days 1 and 8) + 75 mg/m^2^ (day 8) every 3 weeks	4 months (5 cycles)	SD	A: 6 × 4 cm B: 2.4 × 2.6 cm	A: nonmeasurable B: 2.4 × 2.6 cm	Stopped for toxicity (fatigue)
Anastrozole	1 mg po daily	5 months	SD	B: 2.4 × 2.6 cm	B: 14.7 × 10.4 C: 8.7 × 7.5 cm	Stopped for progression
Ifosfamide	9 g/m^2^ IV every 3 weeks	3 months (4 cycles)	SD	B: 14.7 × 10.4 cm C: 8.7 × 7.5 cm	B: 17.7 × 17.6 cm C: 6.5 × 5.2 cm Colonic invasion	Stopped for progression
Lenvatinib + Pembrolizumab	18 mg po daily + 2 mg IV/kg every 3 weeks	8 months (11 cycles)	SD (tumor volume reduction) B: 16.3 × 10.1 cm C: 6.3 × 6.2 cm	B: 17.7 × 17.6 cm C: 6.3 × 6.2 cm	B: 16.8 × 9.1 cm C: 11.2 × 8.1 cm	Arterial embolization added after 6 cycles
Trabectedin	1 mg/m^2^ IV every 3 weeks	2 months (2 cycles)	PD	B: 16.8 × 9.1 cm C: 11.2 × 8.1 cm	Extensive abdominal sarcomatosis	Transition to Palliative Care

## Data Availability

The data presented in this study are available in this article.
